# A Simple and Useful Predictive Assay for Evaluating the Quality of Isolated Hepatocytes for Hepatocyte Transplantation

**DOI:** 10.1038/s41598-019-42720-x

**Published:** 2019-04-16

**Authors:** Muneyuki Matsumura, Takehiro Imura, Akiko Inagaki, Hiroyuki Ogasawara, Kengo Fukuoka, Ibrahim Fathi, Shigehito Miyagi, Kazuo Ohashi, Michiaki Unno, Takashi Kamei, Susumu Satomi, Masafumi Goto

**Affiliations:** 1 Department of Surgery, Tohoku University Graduate School of Medicine, 980-0872 Sendai, Japan; 2 Division of Transplantation and Regenerative Medicine, Tohoku University School of Medicine, 980-0872 Sendai, Japan; 3 Graduate School of Pharmaceutical Sciences, Osaka University, 565-0871 Osaka, Japan

## Abstract

No optimal assay for assessing isolated hepatocytes before hepatocyte transplantation (HTx) has been established, therefore reliable and rapid assays are warranted. Isolated rat hepatocytes were dipped in a water bath (necrosis model), and were also cultured with Okadaic acid (apoptosis model) or vehicle, followed by cellular assessment including trypan blue exclusion (TBE) viability, ADP /ATP ratio, plating efficiency (PE), DNA quantity and ammonia elimination. Hepatocytes were transplanted into the liver of analbuminemic rats, subsequently engraftment was assessed by serum albumin and the histology of transplanted grafts. In the necrosis model, the ADP/ATP ratio was strongly and negatively correlated with the TBE (R^2^ = 0.559, *P* < 0.001). In the apoptosis model, the ADP/ATP ratio assay, PE, DNA quantification and an ammonia elimination test clearly distinguished the groups (*P* < 0.001, respectively). The ADP/ATP ratio, PE and DNA quantity were well-correlated and the ammonia elimination was slightly correlated with the transplant outcome. TBE could not distinguish the groups and was not correlated with the outcome. The ADP/ATP ratio assay predicted the transplant outcome. PE and DNA quantification may improve the accuracy of the retrospective (evaluations require several days) quality assessment of hepatocytes. The ADP/ATP ratio assay, alone or with a short-term metabolic assay could improve the efficiency of HTx.

## Introduction

The first experimental attempt at whole-liver transplantation was initiated in the mid-1950s and the first successful clinical liver transplantation in humans was reported in 1963^1^. Liver transplantation has since become the most successful treatment for the management of end-stage liver disease and acute liver failure but donor shortage is an urgent issue to be solved. The number of patients waiting for a liver transplantation greatly outnumbers the available donors. Even with the expansion to include marginal donors and recent advances in surgical techniques (*i.e*., split-liver and living-related liver donor grafts), there has only been a small increase in the number of donors^2^. Thus, cell-based therapies have been proposed as a solution to some of these issues.

Hepatocyte transplantation (HTx) has been performed as an alternative to liver transplantation to treat lethal acute liver failure^3,4^ and some liver-based inborn errors of metabolism^5,6^. The HTx procedure has several advantages over liver transplantation: (1) HTx (simple injection of isolated hepatocytes into the liver or spleen of the recipients without general anesthesia/laparotomy) is far less invasive than liver transplantation and severely ill patients can be treated with relatively little risk; (2) HTx can be performed repeatedly; (3) hepatocytes from one donor liver may be transplanted into multiple recipients, expanding the donor pool; and (4) the costs should be lower compared to liver transplantation. Despite positive reports and the presumed advantages of clinical HTx, the current literature only includes 143 cases^7–9^. Many challenges remain before HTx can be conducted as a routine clinical treatment.

The poor engraftment of transplanted hepatocytes is one such problem^3,7–9^. Since the first case of clinical human HTx in 1992^10^, HTx has been more successful in animal experiments than in the clinical setting. In many clinical cases, sustained benefits have not been observed; in some cases, HTx had no observable benefit^3^. Poor engraftment may be partly attributed to the induction of apoptosis due to the suboptimal quality of transplanted hepatocytes^11,12^. With the current donor shortage, the main tissue sources are marginal quality livers (*i.e*., steatotic livers, livers from elderly donors, and livers donated after cardiac death that are deemed unsuitable for liver organ transplantation)^13–16^. However, hepatocytes isolated from steatotic livers show low viability and a poor function^17,18^, the quality of hepatocytes isolated from older donors is highly variable^19^, and the viability of hepatocytes from livers donated after cardiac death is low and is well-correlated with the warm ischemic time^20^, clearly indicating that the quality of isolated hepatocytes is a limiting factor for HTx.

Trypan blue exclusion (TBE) is the only established method for evaluating the hepatocyte quality before transplantation in the clinical setting^19,21^. TBE is routinely performed at the end of isolation and before transplantation. TBE reflects the viability based on the membrane integrity and is a simple and rapid method. However, the correlation between TBE viability and the clinical outcomes of HTx is unclear^19,22–25^. In the laboratory setting, studies of short-term drug metabolism and long-term cultured hepatocytes are commonly used to evaluate the quality of transplanted hepatocytes^26–28^. However, considering the fragility of isolated hepatocytes^29^, these assays require relatively too much time to assess the quality of grafts before HTx in the clinical settings; optimal methods must be established.

The quality assessment of graft cells is one of the key issues in the success of pancreatic islet transplantation, which has become an alternative treatment for diabetic patients^30–33^. We previously noted that an adenosine diphosphate (ADP)/adenosine triphosphate (ATP) ratio assay of the isolated pancreatic islets was useful for predicting the clinical outcome of pancreatic islet transplantation^31,34–38^. In contrast, in the field of HTx, no quality assay that is correlated with the outcome of HTx in an *in vivo* model has been developed, whereas several novel assays have been correlated with TBE viability assays^23,26^.

We aimed to establish a rapid predictive assay for evaluating the quality of isolated hepatocytes for HTx. We investigated the ADP/ATP ratio assay and the conventional hepatocyte quality assays (including TBE, plating efficiency [PE], deoxyribonucleic acid [DNA] quantification and an ammonia elimination test) to examine factors associated with the outcome of HTx using an *in vivo* analbuminemic model.

## Results

### Correlation between TBE viability and ADP/ATP ratio in the necrosis model

**Figure 1:**
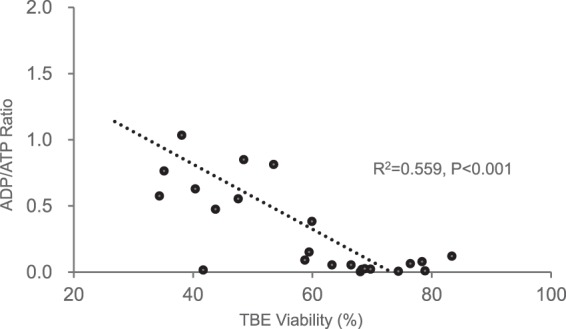
Correlation between trypan blue exclusion (TBE) viability and the ADP/ ATP ratio in the necrosis model The ADP/ATP ratio was strongly and negatively correlated with TBE viability (R^2^ = 0.559, *P* < 0.001). However, our data suggested that the ADP/ATP ratio of hepatocytes of <0.12 (corresponding to TBE viability of > 60%) did not correlate with TBE viability (R^2^ = 0.178, *P* = 0.172) (n = 12).

Heating caused membrane destruction and necrosis in hepatocytes, and subsequently resulted in hepatocytes with variable TBE viability. The ADP/ATP ratio was strongly and negatively correlated with TBE viability (R^2^ = 0.559, *P* < 0.001,). However, our data also suggested that the ADP/ATP ratio of hepatocytes (<0.12; corresponding to a TBE viability of >60%) was not correlated with TBE viability (R^2^ = 0.178, *P* = 0.17) (n = 12) (Fig. 1).

### Evaluation of the hepatocyte viability in the apoptosis model by TBE and the ADP/ATP ratio assay

**Figure 2:**
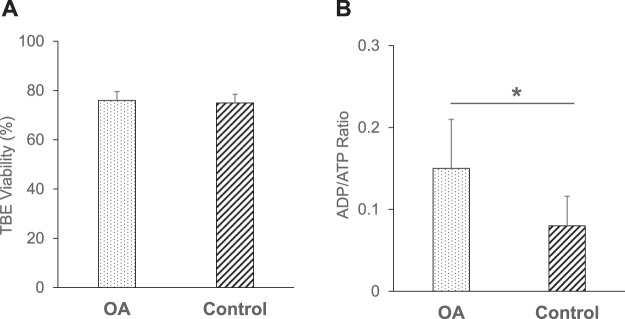
Evaluation of the hepatocyte viability in the apoptosis model by TBE and the ADP/ATP ratio assay. (**A**) The TBE viability of the OA (dotted bar, 75.98 ± 3.59%, n = 18) and control groups (left hatched bar, 74.93 ± 3.50%, n = 18). No significant difference was observed between the OA and control groups. (**B**) The ADP/ATP ratio of the OA group (dotted bar, 0.150 ± 0.060, n = 18) and control group (left hatched bar, 0.080 ± 0.036, n = 18). *P < 0.001 in OA vs. control groups.

To reveal the difference of sensitivity against apoptotic hepatocytes, we evaluated TBE viability and ADP/ATP ratio of hepatocytes in the two groups. The TBE viability of the Okadaic acid (OA) and control groups was 75.98 ± 3.59% and 74.93 ± 3.50%, respectively (n = 18). In terms of TBE viability, no significant difference was observed between the two groups (*P* = 0.582) (Fig. 2A). In contrast, the ADP/ATP ratio of the OA group was significantly higher than that of the control group (0.150 ± 0.060 vs. 0.080 ± 0.036%, n = 18, *P* < 0.001) (Fig. 2B).

### Verification of the apoptotic hepatocytes in the apoptosis model by TUNEL staining and flow cytometry

**Figure 3:**
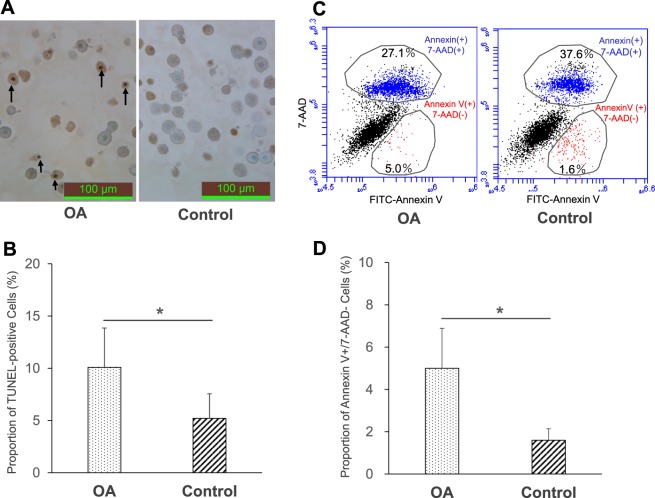
Verification of the apoptotic hepatocytes in the apoptosis model by TUNEL staining and flow cytometry. (**A**) Representative images of TUNEL-staining of the OA and control groups. The nuclei of apoptotic cells were darkly stained and were defined as TUNEL-positive (arrows) (original magnification: 400×, scale bars: 100 μm). (**B**) The proportion of TUNEL-positive cells in the OA (dotted bar, 10.10% ± 3.76, n = 8) and control groups (left hatched bar, 5.20% ± 2.36, n = 8). *P < 0.05 in OA vs. control. (**C**) The percentage of events in the early apoptotic (Annexin V^+^/7-AAD^−^) and late apoptotic (Annexin V^+^/7-AAD^+^) cells are indicated in the schematic illustration. (**D**) The proportion of Annexin V^+^/7-AAD^−^ cells in the OA (dotted bar, 5.0 ± 1.89%, n = 8) and control groups (left hatched bar, 1.6 ± 0.54%, n = 8). *P < 0.001 in OA vs. control groups.

Terminal deoxynucleotidyl transferase-mediated uridine triphosphate nick end labeling (TUNEL) staining and flow cytometry were used to quantify the proportions of apoptotic hepatocytes in the two groups. The proportion of TUNEL-positive hepatocytes in the OA group was significantly higher than that of the control group (10.10 ± 3.76% vs. 5.20 ± 2.36%, n = 8, *P* = 0.028) (Fig. 3A,B). The proportion of Annexin V^+^/7-AAD^−^(apoptotic cells) hepatocytes in the OA group was also significantly higher than that in the control group (5.00 ± 1.89 vs. 1.60 ± 0.54%, n = 8, *P* < 0.001), suggesting that OA could effectively induce early-phase apoptosis in isolated hepatocytes (Fig. 3C,D).

### Evaluation of the hepatocyte viability and function in the apoptosis model by conventional assays

**Figure 4:**
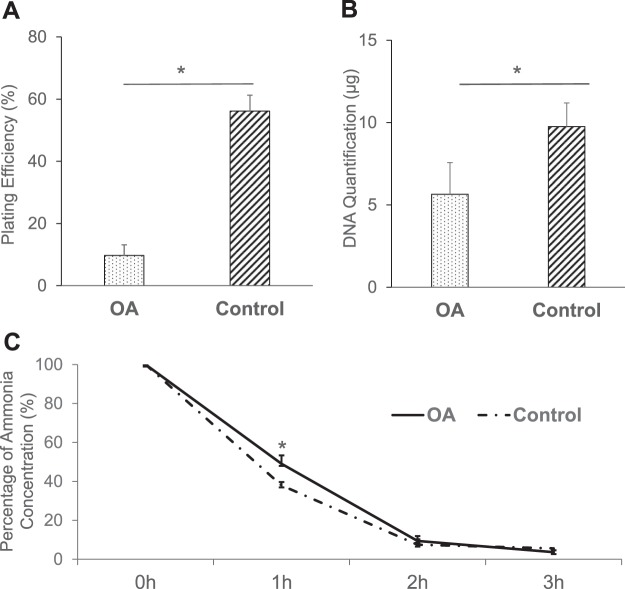
Evaluation of the hepatocyte viability and function in the apoptosis model by conventional assays. (**A**) PE of the OA (dotted bar, 9.72 ± 3.43%, n = 8) and control groups (left hatched bar, 56.13 ± 5.11%, n = 8). *P < 0.001 in OA vs. control. (**B**) DNA quantity at 5 days after the plating of the OA (dotted bar, 5.65 ± 1.92 μg, n = 8) and control groups (left hatched bar, 9.76 ± 1.43 μg, n = 8). *P < 0.001 in OA vs. control. (**C**) The remaining ammonia concentration at 1, 2 and 3-h in the OA (solid line, 49.0 ± 7.22, 9.43 ± 4.34 and 3.66 ± 2.45%, respectively, n = 8) and control groups (dashed line, 37.91 ± 4.06, 7.47 ± 1.77 and 5.60 ± 2.76%, respectively, n = 8). *P < 0.001 at 1-h in OA vs. control.

To investigate whether the conventional assays could effectively distinguish the OA group from the control group, the viability and function in the two groups were examined by PE, DNA quantification and an ammonia elimination test. The PE of the OA group was significantly lower than that of the control group (9.72 ± 3.43 vs. 56.13 ± 5.11%, n = 8, *P* < 0.001) (Fig. 4A). Likewise, the DNA quantity at 5 days after the plating of hepatocytes in the OA group was significantly lower than that of the control group (5.65 ± 1.92 vs. 9.76 ± 1.43 μg, n = 8, *P* < 0.001) (Fig. 4B), while the remaining ammonia concentration at 1 h in the OA group was significantly higher than that in the control group (49.00 ± 7.22 vs. 37.91 ± 4.06%, n = 8, *P* < 0.001). No significant difference was observed in the concentrations at the other time points (2 h; *P* = 0.43, 3 h; *P* = 0.22, respectively) (Fig. 4C).

### The *in vivo* evaluation of transplanted hepatocytes

**Figure 5:**
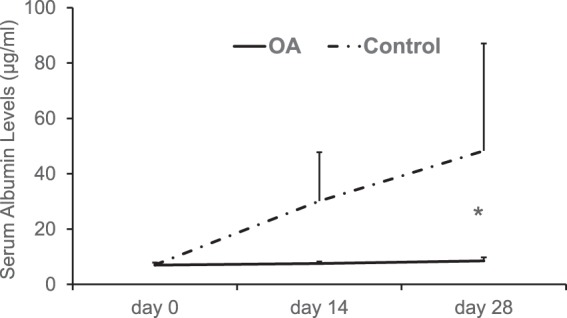
The *in vivo* evaluation of the transplanted hepatocytes from the OA and control groups. The serum albumin levels of the OA and control groups on days 0, 14 and 28 were as follows: OA group, 6.99 ± 0.92, 7.54 ± 0.74, 8.51 ± 1.24 μg/ml, respectively; control group, 7.05 ± 0.82, 30.10 ± 17.64, 48.26 ± 38.75 μg/ml, respectively. *P < 0.05 in OA vs. control.

**Figure 6:**
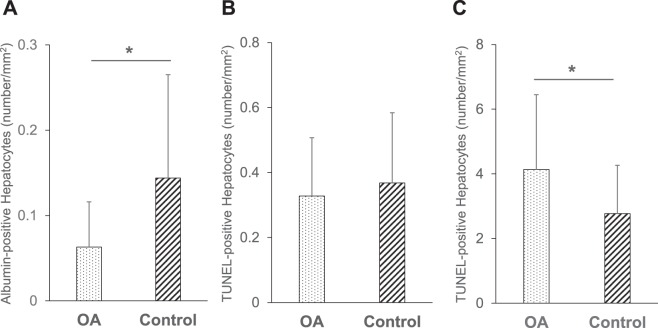
The immunohistochemical analysis of the transplanted hepatocytes from the OA and control groups. (**A**) The number of albumin-positive hepatocytes in the OA (0.063 ± 0.053 cells/mm^2^, n = 8) and control groups (0.144 ± 0.121 cells/mm^2^, n = 8). (**B**) The number of TUNEL-positive hepatocytes in the OA (0.328 ± 0.179 cells/mm^2^, n = 8) and control groups (0.368 ± 0.216 cells/mm^2^, n = 8) on day 28, and (**C**) in the OA (4.136 ± 2.311 cells/mm^2^, n = 8) and control groups (2.769 ± 1.495 cells/mm^2^, n = 8). *P = 0.015 in the OA vs. control groups at 90 min after Htx.

To examine the correlation between several prediction assays and the *in vivo* engraftment efficiency of the transplanted hepatocytes, serum albumin levels were quantified on days 0, 14 and 28 after HTx in analbuminemic rats. The serum albumin levels of the OA group (Day 0: 6.99 ± 0.92 μg/ml, Day 14: 7.54 ± 0.74 μg/ml and Day 28: 8.51 ± 1.24 μg/ml, respectively) were significantly lower than those of the control group (Day 0: 7.05 ± 0.82 μg/ml, Day 14: 30.10 ± 17.64 μg/ml and Day 28: 48.26 ± 38.76 μg/ml, respectively) (*P* = 0.001) (Fig. 5). Although the number of albumin-positive hepatocytes in the OA group (0.063 ± 0.053 cells/mm^2^) tended to be lower in comparison to the control group (0.144 ± 0.121 cells/mm^2^), the difference was not statistically significant (*P* = 0.10) (Fig. 6A). In terms of TUNEL-positive hepatocytes, no significant difference was observed between the OA and control groups on day 28 after HTx (Fig. 6B). By contrast, TUNEL-positive hepatocytes in the OA group were significantly higher than that of the control group (4.136 ± 2.311 vs. 2.769 ± 1.494 cells/mm^2^, n = 7, P = 0.015) at 90 min after HTx.

## Discussion

Our findings revealed that the ADP/ATP ratio of isolated hepatocytes, but not the TBE viability—which is routinely used as quality assay—was correlated with the outcomes of HTx in an *in vivo* model. This is consistent with previous reports which showed that TBE viability measurements were not conclusive enough to predict the outcome of HTx, as there was no correlation between the *in vitro* TBE viability of hepatocytes and the graft function after HTx^19,25^. Both PE and DNA quantification assays were also well-correlated with the *in vivo* performance of the transplanted hepatocyte grafts. Even in the early phase (1 h) of the ammonia elimination assay, a slight tendency toward correlation with the transplant outcome was observed. To the best of our knowledge, this is the first report to show that the results of *in vitro* quality assays of isolated hepatocytes before HTx are well-correlated with their engraftment *in vivo*.

HTx shares many aspects with pancreatic islet transplantation, which is currently regarded as a treatment option for severe diabetes^32^. Considering the similarity between HTx and pancreatic islet transplantation, a number of research experiences based on pancreatic islet transplantation could help to achieve good outcomes in HTx. In the HTx, no assay to predict *in vivo* engraftment before transplantation has been established. In pancreatic islet transplantation, we previously reported that the ADP/ATP ratio of pancreatic islets was strongly correlated with the results of islet transplantation^31^. In that study, we showed that the ADP/ATP ratio, but not the results of current standard quality assays, was correlated with the transplant outcome in diabetic rodents. This is presently considered to be one of the most reliable assays to predict the outcome of clinical pancreatic islet transplantation^39,40^. Hence, in the present study, the ADP/ATP ratio was used to assess the quality of isolated hepatocytes and fruitful results were obtained.

Previous studies showed that the poor engraftment of transplanted hepatocytes was closely related to the apoptotic state of hepatocyte grafts^11,12^. Thus, we created a model of early-phase apoptosis using OA, a fatty acid isolated from the sea sponge (*Halichondria okadaii*), a specific inhibitor of the serine/threonine protein phosphatases (PP) 2A and PP1 that causes the cessation of cell cycle progression^41^, and which was also shown to induce apoptosis in various mammalian tumor cells^42,43^ and isolated rat hepatocytes^42,44^. In this model, TBE could not detect any differences between the OA and control groups, while the ADP/ATP ratio assay clearly identified differences in the hepatocyte quality between the two groups (Fig. 2A,B). In the apoptosis model, TUNEL staining and flow cytometry showed that OA group had a significantly higher fraction of early-state apoptotic hepatocytes (Fig. 3A–D). These data confirmed that the ADP/ATP ratio assay could discriminate between the two groups during early-phase apoptosis. This is most likely explained by the fact that TBE can only detect membrane destruction and cannot detect the apoptotic hepatocytes. In contrast, the ADP/ATP ratio assay detects both apoptotic and necrotic hepatocytes with relatively high sensitivity. We also investigated the conventional assays in this apoptosis model. The PE and DNA quantity of the OA group were significantly lower in comparison to the control group. The remaining ammonia concentration of the OA group at 1 h was also significantly higher than that of the control group; however, the difference was relatively small (Fig. 4A–C). PE and DNA quantification can examine the long-term adherence activity of isolated hepatocytes, and the ammonia elimination test could examine the short-term drug metabolism. One possible explanation for the difference in the data between the long-term adherence activity and the short-term drug metabolism is that the apoptotic hepatocytes may lead to necrosis over a shorter time than we had expected. Although the results of PE and the DNA quantity were well-correlated with the HTx outcome, several days are required before both evaluations can be completed. Given that the isolated hepatocytes are fairly fragile^29^, these assays are not practical for the clinical setting. Alternatively, they may be effective for increasing the accuracy of quality assessment of isolated hepatocytes retrospectively in the research setting. In contrast, both the ADP/ATP ratio and ammonia elimination assays are simple techniques that can rapidly assess the quality of isolated hepatocytes before HTx. In addition, the ADP/ATP ratio assay has several advantages: only a limited number of hepatocytes are required (5 × 10^4^); rapidity (25 min); ease of performance using an established commercial kit; and only standard laboratory equipment is required. Notably, the ADP/ATP ratio assay is remarkably simple and objective in comparison to PE and DNA quantification. The simplicity and objectivity of the ADP/ATP ratio assay make it suitable for use as a predicting assay in the clinical setting.

In the necrosis model, a strong and negative correlation between the TBE viability and ADP/ATP ratio was seen. However, the data also show that the ADP/ATP ratio of hepatocytes <0.12 (corresponding to TBE viability >60%) was not correlated with the TBE viability (Fig. 1), suggesting that the TBE was better for confirming hepatocyte viability in the necrosis model. This is most likely explained by the fact that both ADP and ATP were depleted in the completely destroyed hepatocytes. Therefore, in other word, the ADP/ATP ratio may be particularly useful to predict transplant outcome especially for hepatocytes with small to middle damages.

It is difficult to define the clinical success of HTx. In the present study, analbuminemic rats were used for the *in vivo* assessment of HTx, since this model shows similarities to liver-based inborn errors of metabolism. More importantly, it allows for the effectiveness of engraftment to be quantified. The data indicated that the ADP/ATP ratio was well-correlated with the engraftment of transplanted hepatocytes. In albumin staining, the recipient liver transplanted from the OA group tended to have less engraftment than the control group; however, the result was not statistically significant. TUNEL staining revealed no significant differences between the groups. This is most likely because the apoptotic hepatocytes in the OA group had already disappeared at the time of tissue retrieval. It is speculated that hepatocytes, even at an early phase of apoptosis, are susceptible to cell death due to the inflammation induced by HTx. Thus, one can expect that the highly-sensitive detection of early-phase apoptotic hepatocytes before transplantation would be a powerful tool for predicting the outcomes of HTx. In fact, the present study clearly indicated that the ADP/ATP ratio assay could effectively predict the *in vivo* performance of the transplanted hepatocytes by detecting the proportion of early-phase apoptotic hepatocytes, which can never be discriminated by TBE.

The ADP/ATP ratio assay could accurately detect early-phase apoptotic hepatocytes, is a simple and useful assay that can rapidly predict the outcome of HTx. We propose that the ADP/ATP ratio assay, alone or in combination with metabolic functional assay (*i.e*., the ammonia elimination test) might contribute to improving the efficiency of clinical HTx. This will be validated by further clinical studies using human hepatocytes.

## Materials and Methods

### Animals

Livers were obtained from male inbred F344/NSLc rats (age: 9–10 weeks; weight 180–220 g; Japan SLC Inc., Shizuoka, Japan). The analbuminemic rats (age: 8–10 weeks; weight 160–220 g) were provided by Prof Yuji Nishikawa (Asahikawa Medical College) and were bred at Tohoku University. These analbuminemic rats had a congeneic background to the donor rats^45^. All rats were maintained on 12-h light/dark cycle with *ad libitum* access to food and water. All animals were handled according to the Guide for the Care and Use of Laboratory Animals^46^, and the guidelines for animal experiments at Tohoku University. The experimental protocol of the present study (protocol ID: 2015 NICHe-Animal-001) was approved by the animal experimental committee in the Tohoku University.

### Rat hepatocyte isolation

Rat hepatocytes were isolated and purified using a modified two-step collagenase perfusion technique as previously described^47^. The isolated hepatocytes were suspended in hepatocyte medium (HM) based on Dulbecco’s modified Eagle’s medium (Sigma-Aldrich) supplemented with 10% fetal bovine serum, glutamine, penicillin, streptomycin and 4-(2-hydroxyethyl)-1-piperazineethanesulfonic acid. The yield and viability of hepatocytes were determined by TBE.

### Trypan blue exclusion

The hepatocyte yield and viability were determined by TBE. For this analysis, cell suspension (50 μl) was mixed with 0.4% Trypan Blue (50 μl; Gibco, Waltham, MA, USA). The numbers of dead (blue) and living (white) cells were scored under light microscopy in a Burker chamber (Erma Inc., Tokyo, Japan). TBE viability was expressed as the percentage of living cells in comparison to the total number of cells.

### The ADP/ATP ratio assay

An ApoSENSOR ADP/ATP Ratio Bioluminescent Assay kit (Biovision, San Francisco, CA, USA) was used to measure ADP/ATP ratio. This kit detects the ATP level based on bioluminescence via a luciferase catalyzed reaction. The ADP level is measured by its conversion to ATP catalyzed by ADP converting enzyme. We modified the manufacturer’s instructions and optimized the protocol to specifically measure the viability of hepatocytes. Briefly, 5 × 10^4^ hepatocytes were suspended in a single tube, then mixed with nucleotide releasing buffer (200 μl) for 10 min at room temperature. Thereafter, ATP monitoring enzyme (10 μl) was added to the solution, and the ATP level was measured using a GloMax-20/20 Luminometer (Promega, Madison, WI, USA) and expressed as the number of relative light units (RLU). After 10 min, the ADP in the solution was converted to the ATP by adding of ADP converting enzyme (10 μl), and the RLU was measured immediately before and after 5 min of conversion. Subsequently, the ADP/ATP ratio of the hepatocytes was calculated as (C-B)/A, where A is the RLU for ATP, B is the RLU of ATP immediately before conversion from ADP, and C is the RLU of ATP at 5 min after conversion from ADP; the evaluation was completed in 25 min.

### Induction of necrosis by heating

We evaluated the efficacy of the ADP/ATP ratio assay in assessing hepatocyte viability in a necrosis model. Immediately after hepatocyte isolation, 2.5 ml of suspended hepatocytes (5 × 10^5^ cells/ml) was placed in a single tube and dipped in a 60 °C water bath for 2 or 3.5 min. Then, aliquots of suspension were used for TBE and the ADP/ATP ratio assay (n = 12).

### Induction of apoptosis using Okadaic acid

We evaluated the efficacy of ADP/ATP ratio assay in assessing the viability of hepatocytes in the apoptosis model using OA (Wako, Osaka, Japan). Two milliliters of hepatocytes (5 × 10^5^ cells/ml) were seeded on non-treated 35-mm petri dishes (Asahi glass, Tokyo, Japan). The cells were cultured in HM supplemented with 100 nmol/L OA dissolved in dimethyl sulfoxide (DMSO) (OA group), or with only DMSO (control group). Both of the cells were cultured for 100 min at 37 °C in a humidified atmosphere with 95% air and 5% CO_2_. The final DMSO concentration was 1% in both groups. Both groups were washed twice and re-suspended in HM. Aliquots of each cell were used for TBE and the ADP/ATP ratio assay (n = 18).

### Terminal deoxynucleotidyl transferase-mediated uridine triphosphate nick end labeling (TUNEL) staining

In both groups 2 ml of cells (5 × 10^5^ cells/ml) were fixed, embedded in paraffin and prepared on glass slides. TUNEL staining was performed with a TACS2 TdT DAB kit (Trevigen, Gaithersburg, MD, USA), according to the manufacturer’s instructions. Apoptotic cells were indicated by dark brown staining of the nucleus. The percentage of apoptotic cells was estimated by counting the number of the apoptotic cells in comparison to the total number of cells in 10 random microscopic fields (n = 8).

### Flow cytometry

Flow cytometry was performed in the two groups. Briefly, 2 ml of hepatocytes (5 × 10^5^ cells/ml) was washed and re-suspended in 1 ml of 1 × Binding Buffer (BD Biosciences, Franklin Lakes, NJ, USA) and stained with Annexin V (BD Biosciences) and 7-AAD (BD Biosciences) for 15 min in the dark at room temperature. Each cell was analyzed on an Accuri C6 Flow Cytometer (BD Biosciences); 5,000 cells were acquired. The vital cells were Annexin V^−^/7-AAD^−^, the apoptotic cells were Annexin V^+^/7-AAD^−^, and the necrotic cells were Annexin V^+^/7-AAD^+^ (n = 8).

### Evaluation of the hepatocyte viability and function in the apoptosis model by conventional assays

The viability and function of the OA and control groups were examined according to the PE, DNA quantification and an ammonia elimination test. Two milliliters of cells from each group (5 × 10^5^ cells/ml) was seeded on type I collagen-coated 35-mm petri dishes (AGC Techno Glass, Shizuoka, Japan). Each group was cultured in HM at 37 °C in a humidified atmosphere with 95% air and 5% CO_2_. The PE was determined by counting the attached and unattached cells using a light microscope (×200 magnification) at 18 h after plating. The PE of each group was expressed as the percentage of attached hepatocytes in comparison to the total number of plated hepatocytes. Six fields were randomly studied for each culture dish (n = 8). The HM was changed on days 1 and 3. DNA quantification was performed to evaluate the energy status of the cultured hepatocytes at 5 days after plating. The DNA content was measured using a DNA quantification kit (Primary cell, Ishikari, Japan) twice for each dish, as previously described (n = 8)^36,48^. Ammonia-load culture medium was prepared with a known concentration of ammonium chloride solution and Williams Medium E containing 10% fetal bovine serum, 1 μmol/L insulin, and 1 μmol/L dexamethasone. The concentration of ammonia was adjusted to approximately 2.2 mmol/L. In each group, 1 × 10^6^ hepatocytes in 1 ml of ammonia-load culture medium were cultured using non-treated 35-mm petri dishes for 3 h at 37 °C in a humidified atmosphere with 95% air and 5% CO_2_. The concentration of ammonia at 0, 1, 2, and 3 h was measured in 20 μl of the ammonia-load culture medium using an Amicheck Meter (Arkray, Inc., Kyoto, Japan). The ammonia concentration at each point was shown as the percentage in comparison to the concentration at 0 h (n = 8)^49^.

### The *in vivo* evaluation of the transplanted hepatocytes from the OA and control groups

The viability and functions of the OA and control groups were examined by the *in vivo* albumin production. Ten million hepatocytes from the OA and control groups were spun down and pellets (200 μl) were directly injected into the portal vein of analbuminemic rats using a 25-G needle with a gastight syringe (Hamilton Company, Reno, NV, USA) for 60 s^50,51^. Blood samples were taken on days 0, 14, and 28. The serum albumin levels were quantified using a Rat Albumin ELISA kit (AKRAL-120, Shibayagi, Gunma, Japan). On day 28, the recipients were sacrificed and hepatic tissues were retrieved. The right, left, quadrate and caudate lobes of each sample were prepared, and albumin staining was performed using anti-albumin antibodies (MP Biomedicals, Santa Ana, CA, USA) combined with the VECTASTAIN ABC system (Vector Laboratories, Inc., Burlingame, CA, USA). At 90 min and day 28 after HTx, TUNEL staining was performed on hepatic tissues using a TACS2 TdT DAB kit (Trevigen). The number of albumin-stained and TUNEL-positive hepatocytes in each sample was counted by microscopy and divided by the estimated area (n = 8).

### Statistical analysis

All values were expressed as the means and standard deviation. The statistical analyses were performed using the JMP pro 12 software program (SAS institute Inc., NC, USA). Student’s *t*-test was used for comparisons between the two groups. The serum albumin levels were analyzed by a two-way ANOVA and a Tukey-Kramer test was used for post-hoc comparisons between groups. The relationship between TBE viability and the ADP/ATP ratio in the necrosis model was analyzed with Pearson’s correlation coefficient. *P* values of < 0.05 were considered to indicate statistical significance.
